# EARLY ADOPTION AND LONG-TERM USE: ANALYZING OLDER ADULTS’ USAGE PATTERNS AND PREDICTORS OF PRISM GERMANY

**DOI:** 10.1093/geroni/igae098.0042

**Published:** 2024-12-31

**Authors:** Nicole Memmer, Anna Schlomann, Hans-Werner Wahl, Leon Radeck

**Affiliations:** Heidelberg University, Heidelberg, Baden-Wurttemberg, Germany; Network Aging Research, Heidelberg, Baden-Wurttemberg, Germany; Heidelberg University, Heidelberg, Baden-Wurttemberg, Germany; Institute of Computer Science, Heidelberg, Baden-Wurttemberg, Germany

## Abstract

This study introduces PRISM-Germany, the German version of the PRISM-App originally developed by North American research consortium CREATE (Czaja et al. 2018), aiming to support social participation among older adults. Addressing the challenge of long-term adherence, we explored changes in app usage over 3 months and identified predictors of use, focusing on socio-demographic and technology-related variables. The analytical sample consisted of 171 community-dwelling adults aged 67–93 (mean age=73.7 years; female=55%) with medium digital competencies as part of the ongoing SMART-AGE complex intervention trial. Usage behavior in PRISM-Germany was assessed through a cloud-based data log procedure, providing real-time recording of individual usage behaviors. A baseline data protocol collected data on relevant predictors via the Redcap online software. Usage analyses indicated that on average 11 out of 32 available categories (e.g. email, video-chat, health information) were accessed in PRISM-Germany per week. A significant decline in average usage over time was observed, ranging from 1st month: m = 15.3 to 2nd month: m = 9.7, and finally to 3rd month: m = 7.5. ANCOVA revealed the decline as robust, controlling for socio-demographic and technology-related variables. At the multivariate level, chronological age, health, general technology acceptance, and previous technology competencies did not predict frequency of use, while female gender, not living alone, and being lower educated did (Adjusted R2 = 0.108). Concluding, 50% reduction in app use across three months as well as the sole predictive role of sociodemographic factors seem remarkable.

